# Supplementary material for: Modulation of intestinal bile acids influences colonic mucosal responses
Source: Sci Rep. 2026 Jun 3;16:17126. doi: 10.1038/s41598-026-55206-4 (PMC13234397; doi:10.1038/s41598-026-55206-4)
Supplement: Supplementary file 2 — Supplementary Information 2. [file 41598_2026_55206_MOESM2_ESM.docx]

**Supplementary Table S1** Significant genes per cell cluster

**Supplementary Figure S1** Descriptive scRNA-Seq data on quality

**Supplementary Figure S2** Experimental scheme of the work with tissue microarrays (TMAs). Abbreviations: ID, identity; FFPE, formalin-fixed paraffin-embedded.

**Supplementary Figure S3** Volcano plot of differently expressed genes in the gut epithelium of mice colonized with the Oligo-Mouse Microbiota 12 (O) without or with addition of the deoxycholic acid (DCA)-producing species *Extibacter muris* (E). The data is shown per cell cluster.
