# Supplementary figures and images for: Modulation of intestinal bile acids influences colonic mucosal responses
Source: Sci Rep. 2026 Jun 3;16:17126. doi: 10.1038/s41598-026-55206-4 (PMC13234397; doi:10.1038/s41598-026-55206-4)

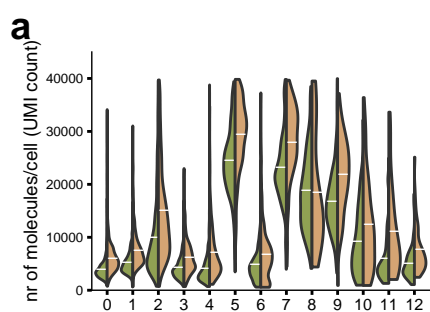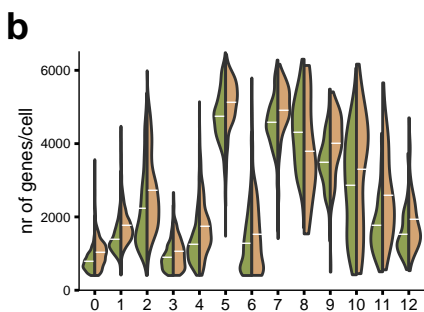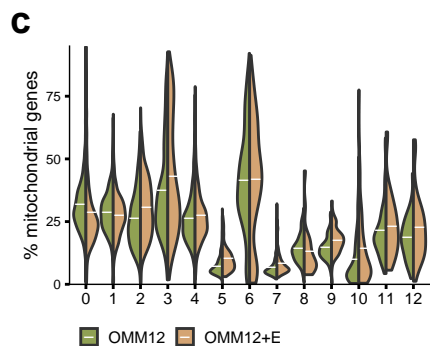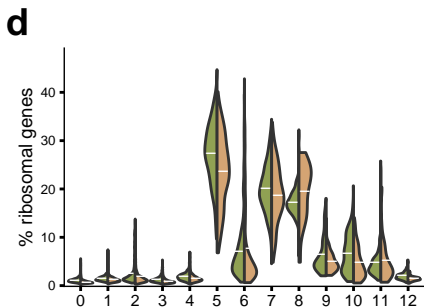

Supplement: Supplementary file 5 — Supplementary Information 5. [file 41598_2026_55206_MOESM5_ESM.pdf]

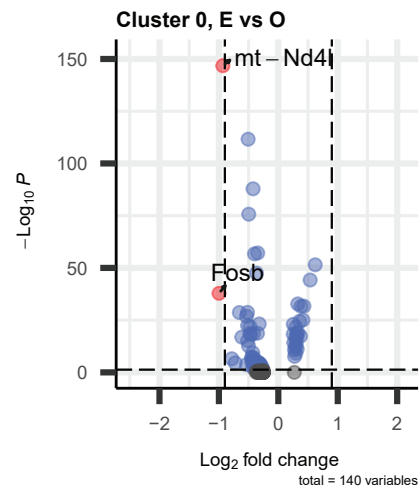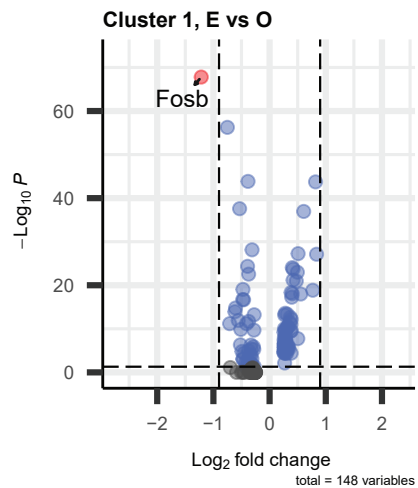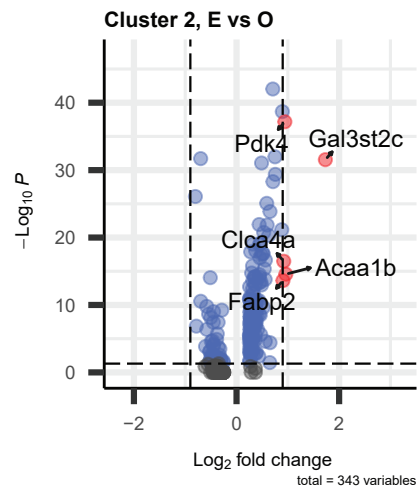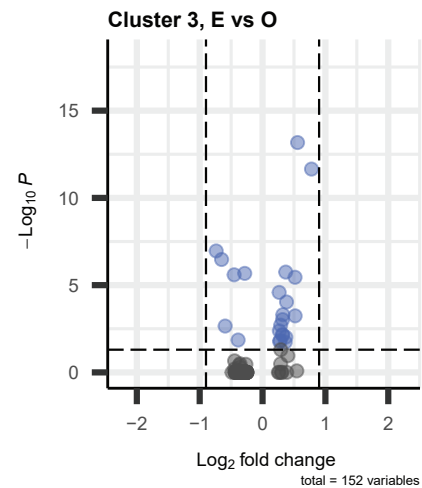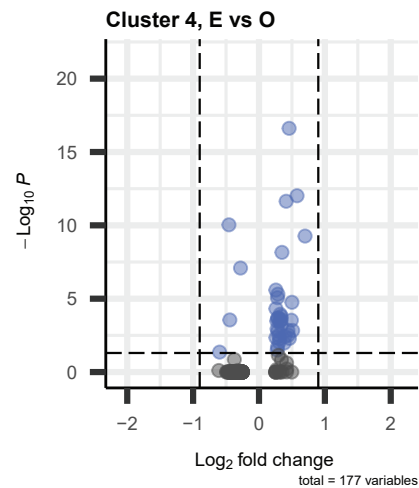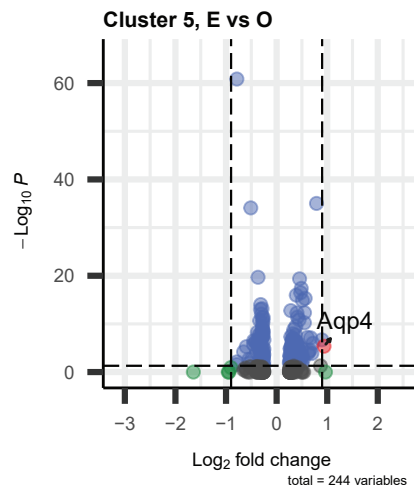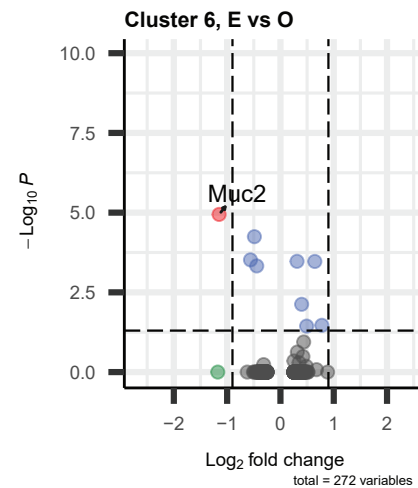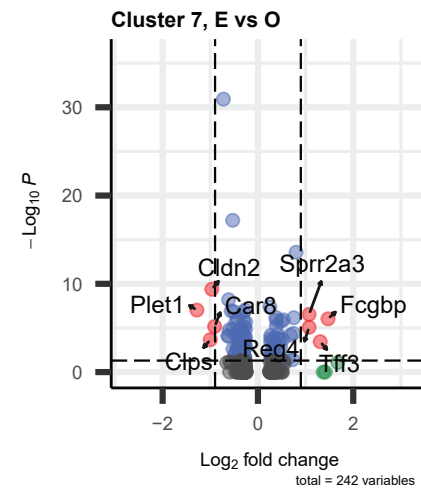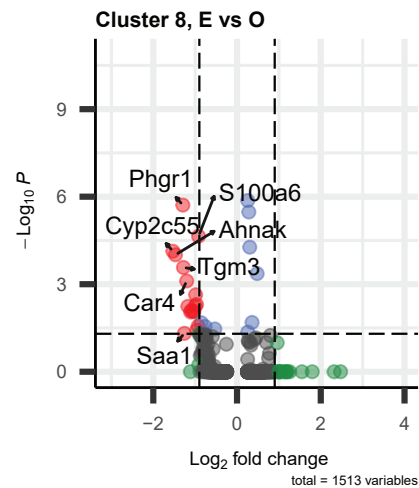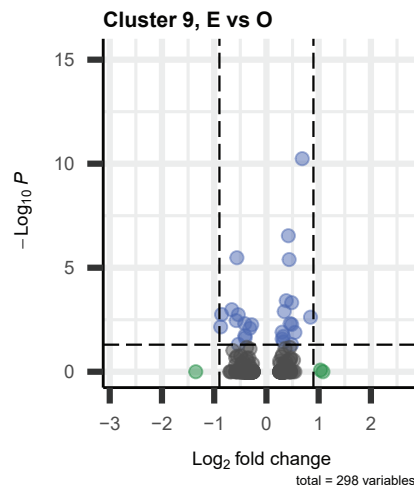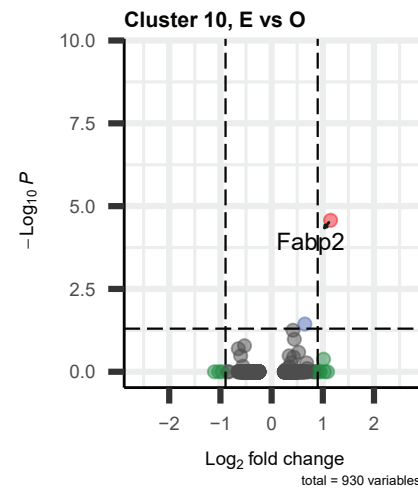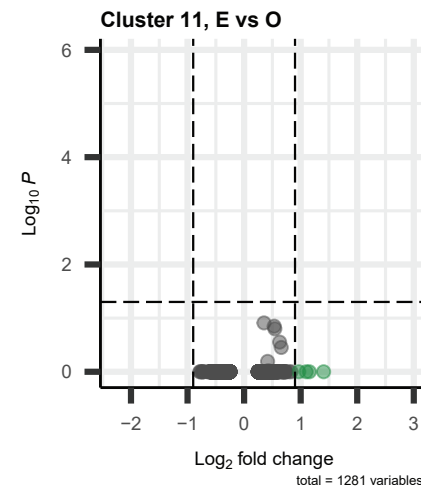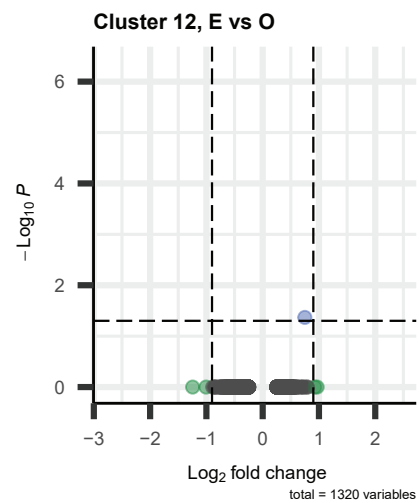

● NS  
● Log2FC  
● p.adj  
● p.adj & Log2FC

Supplement: Supplementary file 7 — Supplementary Information 7. [file 41598_2026_55206_MOESM7_ESM.pdf]
