# Supplementary material for: Modulation of intestinal bile acids influences colonic mucosal responses
Source: Sci Rep. 2026 Jun 3;16:17126. doi: 10.1038/s41598-026-55206-4 (PMC13234397; doi:10.1038/s41598-026-55206-4)
Supplement: Supplementary file 6 — Supplementary Information 6. [file 41598_2026_55206_MOESM6_ESM.pdf]

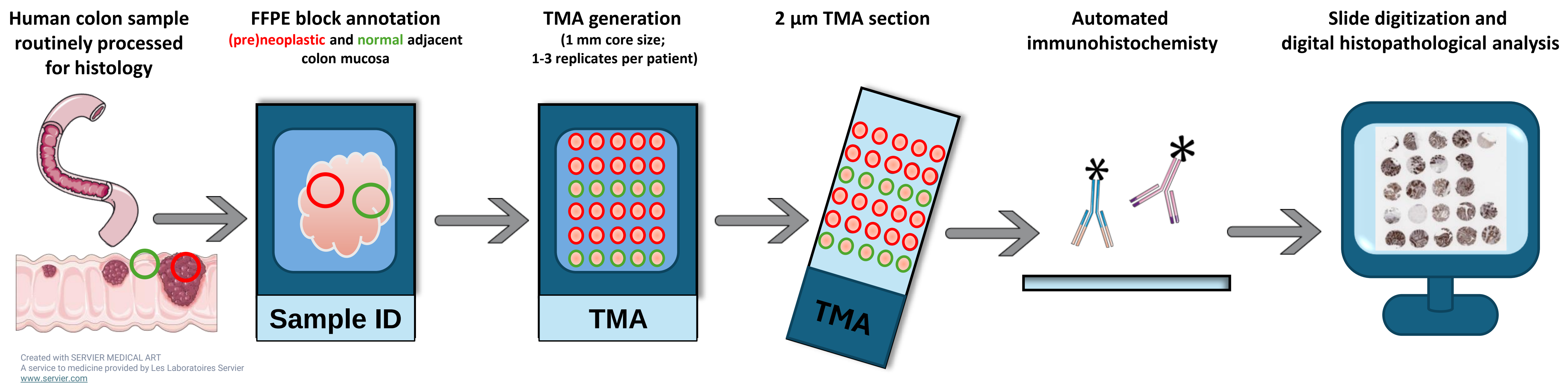

Human colon sample routinely processed for histology
FFPE block annotation
(pre)neoplastic and normal adjacent colon mucosa
TMA generation
(1 mm core size;
1-3 replicates per patient)
2 µm TMA section
Automated immunohistochemisty
Slide digitization and digital histopathological analysis
Sample ID
TMA
TMA
Created with SERVIER MEDICAL ART
A service to medicine provided by Les Laboratoires Servier
www.servier.com
